# Effects of a systematically offered social and preventive medicine consultation on training and health attitudes of young people not in employment, education or training (NEETs): An interventional study in France

**DOI:** 10.1371/journal.pone.0216226

**Published:** 2019-04-26

**Authors:** Sarah Robert, Lucile Romanello, Sophie Lesieur, Virginie Kergoat, Joël Dutertre, Gladys Ibanez, Pierre Chauvin

**Affiliations:** 1 INSERM, Sorbonne Université, Institut Pierre Louis d'Epidémiologie et de Santé Publique, Department of Social Epidemiology, Paris, France; 2 Sorbonne Université, Pierre et Marie Curie Faculty of Medicine, Department of Education and Research in General Medicine, Paris, France; 3 INSERM, UVSQ, Université Paris-Saclay, Population-Based Epidemiological Cohorts Unit (UMS 11), Villejuif, France; 4 Mission locale de Sénart, Lieusaint, France; Northumbria University, UNITED KINGDOM

## Abstract

**Background:**

NEETs (young people not in employment, education or training) are at higher risk for poorer mental and physical health. In France, the *Missions locales* (MLs) are the only social structures dedicated to this population. We sought to determine whether the systematic offer of a social and preventive medicine consultation at a ML might increase NEET participants’ access to training in the 12 months following the intervention.

**Methods:**

This intervention research was a parallel randomised controlled interventional study conducted at five MLs in mainland France in 2011–2012. It included 976 NEETs aged 18 to 25 years who attended one of the five MLs. At inclusion, participants were randomly assigned (1:1:1) to three groups: those in the first group were invited to see a social worker (not studied in this paper), those in the second group were invited to see a doctor and a social worker (intervention group), and the third was a control group. The primary outcome was participation in at least one training session during the year following study inclusion.

**Results:**

Among the 976 participants, 504 were randomly assigned to the intervention group and 472 to the control group; 704 (72.1%) were included in the analyses. A significantly higher proportion of the participants in the intervention group participated in a training session in the 12 months following the intervention than of those in the control group (63.3% *vs* 55.6%; p = 0.04). This difference was significantly greater for women, those less than 21 years of age, those unstably housed and those with a lower level of education.

**Conclusions:**

Social and preventive medicine consultations that are fully integrated into the social services for NEETs have an impact on their access to training and contribute to changing some of their health-related behaviours. This may improve their access to the labour market.

## Introduction

The NEET concept refers to a specific subgroup of young people that are not in employment, education or training. This acronym appeared first in the United Kingdom in the 1980s [1]. In 2010, the European Commission adopted the proportion of NEETs in the population of young people aged 15 to 29 years as an indicator of their integration into the job market. According to Eurofound data, the proportion of NEETs has continued to increase in the past few years, including in France, essentially because of the negative effects of the 2007/2008 economic crisis on employment [2]. In 2012, they accounted for 15% of the 15- to 29-year-olds in France [3]. In certain underprivileged areas, this proportion can be as high as 30% [4]. Research suggests that spending time in NEET status at a young age can have long lasting consequences or ‘scars’. These scars can have a negative effect on future employment outcomes and earnings as well as negative consequences on physical and mental health [1]. Social health inequalities apply to adolescents and young adults, too [5–7]. In the United Kingdom and Finland, Rahkonen and collaborators report that the level of education and social class are two main factors that explain health differences in young adults [8]. In addition, certain international publications have highlighted the link between occupational inactivity and health problems in young people, whether it is in France, Poland, Spain, Sweden, Switzerland or the United Kingdom, but also in Australia and in Canada [9–17]. In Sweden, Helgesson and collaborators observed an increased risk of mortality, of being out of work for a prolonged period of time or of receiving a disability pension during the 15 years following a period of unemployment in young people aged 20 to 24 years (compared to those of the same age who were not unemployed) [18].

In France, the 440 *Missions locales* (MLs), which operate throughout the country, perform a public service mission at the neighbourhood level to enable young people aged 16 to 25 years who have no training and no job to overcome the difficulties that impede their social and occupational integration [19,20]. They are the only facilities dedicated to this population that have been established in France. They are charged with welcoming, informing, providing vocational guidance to, and supporting young people in helping them build a career and life plan. The central concept of MLs is the global approach, that is, the inseparability of the work and social dimensions: making all efforts to facilitate young peoples’ access to jobs and independence, especially through individual follow-up by a counsellor (who generally have training as social workers).

The population targeted by MLs consists of NEETs aged 16 to 25 years. In 2013, more than 1.5 million young people in difficulty visited an ML in France at least once, and the MLs received an average of ten to fifteen percent of all young people in their respective territories. The original mission of MLs, which were created in 1982, was to attend to the training, employment, housing and health of these young people. Little by little, with the continuous rise in unemployment among the young people concerned, the public authorities ordered the MLs to concentrate essentially on access to jobs and training.

Originally, at certain MLs, there were social and preventive medicine consultations whose objectives were to promote health, the use of appropriate care, and social integration. These consultations helped to identify and refer young people who needed primary health care to the usual health care system (especially in general medicine). They were most often far removed from the existing offer of primary care because of their age and difficult social situations. With time, these social and preventive medicine consultations disappeared at most MLs. The social workers dedicated to access to health insurance under Social Security and the physicians who were working at the few MLs that were still providing these services therefore asked our research team to determine if their presence among the MLs had added value, not only in terms of health promotion, but also in terms of access to training and jobs. We conducted a multicenter interventional research project aimed at determining if systematically offering a social and preventive medicine consultation to NEETs who frequented five MLs would increase their participation in training sessions during the year following the intervention, knowing that there is an important link between training and job access, especially since a low level of education is the key risk factor for being a NEET.^1^ Given the length, complexity and diversity of the different types of job access (work term, interim, short contracts, subsidized contracts, etc.), it seemed difficult to make them a primary outcome. However, access to work within the year following the intervention was a secondary outcome.

## Materials and methods

### Study design and participants

PRESAJE (the French acronym for a research project on young people’s health) was an unmasked, randomised controlled parallel interventional study conducted in five French cities in 2011–2012.

Participants were recruited from five selected MLs in Clichy, Sénart, Toulouse, Poitiers, and Reims. Clichy, Sénart and Toulouse are in urban areas (the first two cities are in the Paris metropolitan area and the last one is a regional capital), while Poitiers and Reims are both medium-sized towns in rural areas. These facilities were selected on the basis of their interest in the study and their ability to implement its empirical design. Because one treatment consisted of the intervention by health professionals, only MLs with at least a part-time general practitioner (GP) in-house could take part in the study.

All young people aged 18 to 25 years who came to one of these five facilities a second time were invited to participate. Since more than half of the young people who visit an ML do so only once (e.g., only to obtain information), we decided to select only those who were likely to avail themselves of the services–that is, to undergo long-term counselling and a follow-up by a counsellor–in order both to select the real target population of recipients of MLs’ services and to reduce attrition bias. After their meeting with their counsellor, the young people were sent to the member of the field staff in charge of presenting the study design. They could choose to agree or not agree to participate. Participants were ineligible if they were unable to speak or understand French because the consent forms and questionnaire were provided only in French. The study protocol was approved by the French authorities: the *Comité consultatif sur le traitement de l’information en matière de recherche dans le domaine de la santé* (CCTIRS) and the *Commission nationale de l’informatique et des libertés* (CNIL) (authorisation number 1527880), in accordance with French legislation. All the patients gave their written informed consent. This study was listed on the ISRCTN registry with study ID ISRCTN59210540.

### Randomisation and masking

Patients were recruited between January 3, 2011 and January 2, 2012. They were randomised upon the receipt and signing of the informed consent form and the baseline questionnaire. Research assistants were in charge of assigning the participants to the study groups (1:1:1) using a computer-generated random list in the order of their inclusion. Neither the participants nor the investigators were masked to group assignment in this open trial.

### Procedures

This study is part of a trial that includes three groups. Eligible participants were randomly assigned to one of three groups (1:1:1): treatment group 1, treatment group 2 or the control group.

The participants in treatment group 1 were systematically invited to an interview with a social worker (social worker group). The main objective of this treatment was to reduce or eliminate financial barriers to access to health care. Taking into account the participants’ then-current situation regarding their health insurance status, the social workers were in charge of providing basic information about the French health-care and insurance systems, finding the most advantageous coverage for them, and assisting them in registering with Social Security for their basic health insurance and in obtaining supplemental health insurance.

In treatment group 2 (the intervention group in the rest of the paper), the participants were additionally encouraged to consult with a doctor for an on-site social and preventive medicine consultation. The purpose of these consultations with a GP was to investigate the young person's health status and health-care habits and practices, to provide them with health information (sexual health, health risk behaviours, healthy lifestyles), and to refer them to health-care services, if warranted. The main goals of this second treatment were to detect serious health problems, encourage healthy behaviours, and increase the participants’ autonomy in managing their health and health care. The consultation practices and contents were neither systematic nor formally standardized, but the five GPs involved in the project met once to be reminded of the objectives of the consultations and to enable them to share their professional approaches and practices with the consultants. No reminder to attend the social and preventive medicine consultation was done. Participants could attend these social and preventive medicine consultations as many times as they wanted.

The third group was the control group. The participants in this group received the usual social services offered by the MLs as those in the two experimental groups. They were not encouraged to meet with a doctor or social worker, but when necessary, they could make an appointment with one (in these cases, they were mainly referred by their counsellor).

The interventions for participants of treatment group 1 (the invitation to see a social worker) and treatment group 2 (the invitation to attend social and preventive medicine consultations) were proposed just after randomisation. The participant had one year to see the professionals.

Demographics, socioeconomic characteristics and certain health data were collected at inclusion using a face-to-face questionnaire administered by research assistants. One year later, the participants were contacted again by phone and invited to an interview with the same research assistant, who completed a final questionnaire. No major protocol changes were made to the inclusion criteria or the treatment interventions during the study. The five research assistants (one per location) met six times in total in order to standardize the interventions and to complete the baseline and final questionnaires in a similar manner.

When the study was designed, we initially wanted to study the effect of a social and preventive medicine consultation, as described in the introduction. An economist suggested that we add a third group to the study (the social worker group) to study the unique effect of reducing or eliminating financial barriers to access to health care. In practice, we observed a high variability in terms of health insurance (in Clichy’s ML, all the participants could very easily pretend to have complete health coverage because a local project was being tested), but also in terms of practices of the social workers. Furthermore, doctors and social workers worked together, and the need felt by young people to get the best health coverage was very low as soon as they assumed they were in good health. The social worker intervention gained meaning after the visit to the GP. Consequently, we assumed that the multi component intervention would be more effective. Therefore, we present in this paper the comparison between the intervention group and the control group to answer our main objective about the combined physician/social worker intervention. However, because the study was designed with the three groups, the methodology as well as the description of the trial profile are made with the three groups. The rest of the results are presented only for the intervention and control groups.

### Outcomes

The primary outcome was participation in a training session during the year following study inclusion. Other outcomes included access to employment (having worked) during the year following study inclusion, and certain characteristics related to health status (perceived health status grouped into two categories: very good or good, versus average, poor or very poor), health care (having: basic health insurance provided by Social Security; supplemental health insurance; a regular GP; seen a psychologist in the last year; and had unmet health needs in the previous year), health knowledge (assessed by the correct [true/false] answers to the following statements: “The morning-after pill is the pill taken the day after missing a birth control pill.” and “The morning-after pill protects against AIDS and sexually transmitted infections.”), and health-related behaviours (assessed by the questions “Do you do anything to maintain your health. If so, is it through diet? Through exercise?”, and by the reporting of no contraceptive method having been used during the last sexual intercourse, including condoms by men).

### Statistical analysis

A preliminary study had estimated that 55% of the young people who visited the MLs participated in a training session during the year following the start of their follow-up. To detect a 10-point increase for each of the interventions in relation to the control group, it was estimated that 409 patients were needed in each group with a power of 90% and a type I (alpha) risk of 0.05 (one-sided test). The theoretical lost to follow-up rate was estimated at 15%. It was therefore necessary to include 481 patients in each group.

The data analyses were performed on the intent-to-treat population (except for certain results specifically mentioned as being per-protocol).

Comparisons between the intervention group and the control group mainly used χ^2^ tests (or Fisher exact tests when the sample sizes were too small) to compare:

- the intervention and control groups at baseline;
- the follow-up and lost to follow-up participants’ characteristics at baseline;
- the intervention and control groups 12 months after inclusion in the intent-to-treat analysis for all participants and by subgroups by gender, age group, type of housing, and level of education at baseline;
- the intervention and control groups 12 months after inclusion in the intent-to-treat analysis by centers (ie in each ML), but also regrouping the MLs into two subgroups (urban MLs *vs* medium-sized towns MLs).
- the intervention group participants who actually saw a doctor and control group 12 months after inclusion (actually for only one outcome: to have seen a psychologist in the last year).

The Student’s *t*-test was used for means comparisons, where appropriate.

Outcome differences between intervention and control groups were computed in two different manners: one, as absolute prevalence differences between the two groups at 12 months (when data had been collected only at 12 months but not at inclusion); the other (when data had been collected both at inclusion and at 12 months), as a difference in the proportion of people with favourable changes between the two groups (difference of differences approach).

The statistical analyses were performed using Stata/SE 12.1. A p-value <0.05 was considered significant.

CONSORT 2010 checklist of information to include when reporting a randomised trial can be found in Appendix 1.

## Results

**Fig 1:**
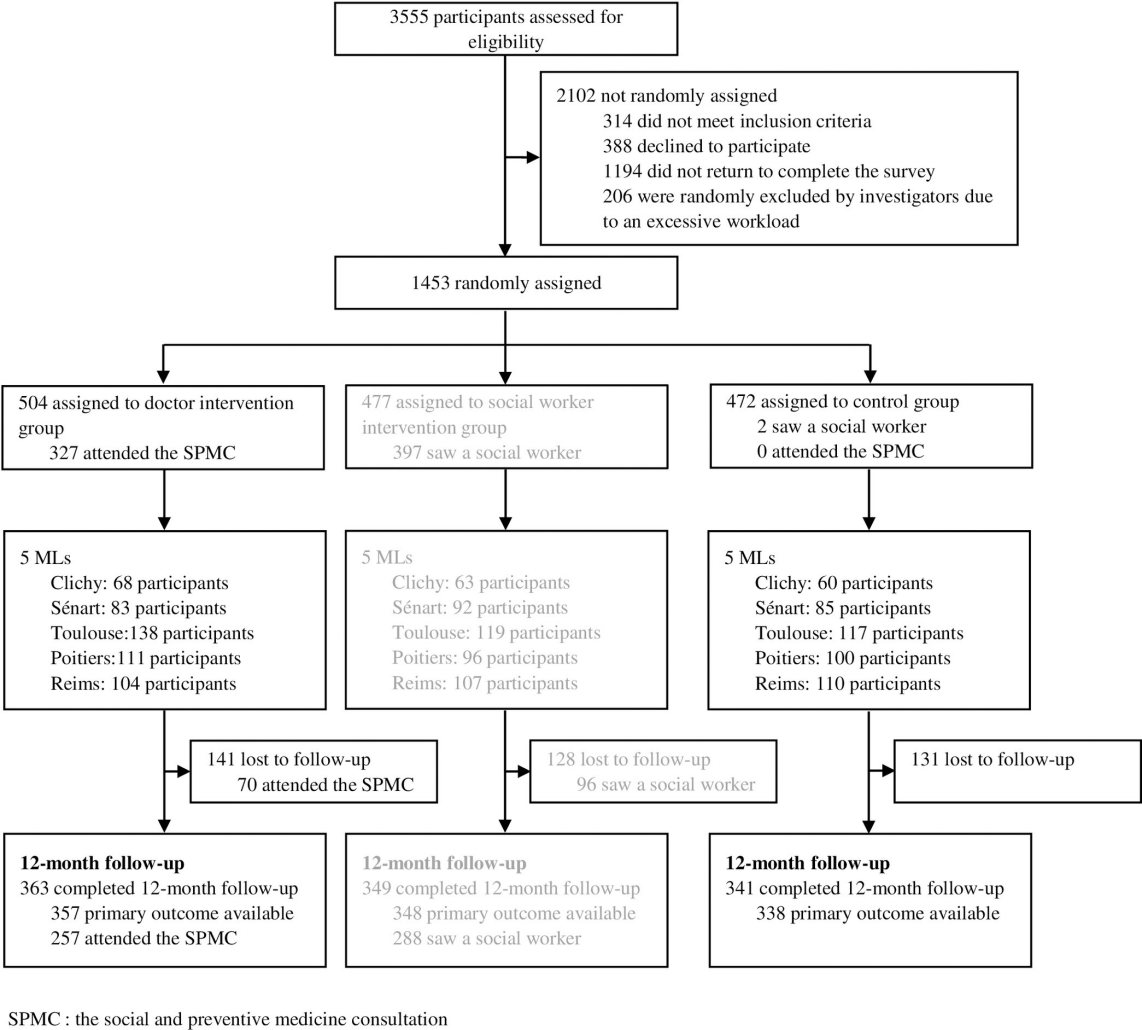
Trial profile.

Of the 3555 eligible individuals, 2102 (59.1%) were excluded: 314 (8.8%) did not meet the inclusion criteria (73 were under 18 years of age, two were over 26 years of age, 239 did not speak French well enough to answer the questionnaires), 388 (10.9%) declined to participate, 206 (5.8%) were excluded by the investigators due to excessive workload, and 1194 (33.6%) did not return to complete the baseline questionnaire (Fig 1). The remaining 1453 (40.9%) individuals were enrolled and randomly assigned: 504 (34.7%) to the intervention group, 477 (32.8%) to the social worker group, and 472 (32.5%) to the control group. Of those enrolled, 363 from the intervention group, 349 from the social worker group and 341 from the control group completed both the baseline and 12-month questionnaires, for a completion rate of 72.4%.

The intervention and control groups have similar characteristics (Table 1). At baseline, the mean age of everyone in the study was 21.3 years (SD: 1.98). Just over half were female (52.2%); 64.3% were French, born to two French parents; 26.5% were French, born to at least one foreign parent; and 9.1% were foreign immigrants. Three-quarters (74.9%) had not continued their education beyond high school, and 15.0% were unstably housed. The participants lost to follow-up were in a more disadvantaged situation than those reinterviewed at 12 months (S1 Table). The lost to follow-up rate was similar in the intervention and control groups (28.0% *vs* 27.8%, respectively; p = 0.94). The participants lost to follow-up’ characteristics were similar in both groups (S2 Table).

**Table 1:** Baseline characteristics.

	Intervention group (n = 504)	Control group (n = 472)	*p*
Age (years)	21.3 (1.9)	21.2 (2.1)	*0*.*42*
Gender			*0*.*21*
Female	253 (50.2%)	256 (54.2%)	
Male	251 (49.8%)	216 (45.8%)	
Origin			*0*.*61*
French, born to two French parents	317 (62.9%)	311 (65.9%)	
French, born to foreign parent(s)	140 (27.8%)	119 (25.2%)	
Foreigner	47 (9.3%)	42 (8.9%)	
Level of education			*0*.*36*
Middle school	40 (8.0%)	48 (10.2%)	
High school	329 (65.5%)	312 (66.1%)	
Postsecondary	133 (26.5%)	112 (23.7%)	
Difficulty reading French*	81 (16.1%)	75 (15.9%)	*0*.*93*
Difficulty writing in French*	144 (28.6%)	151 (32.0%)	*0*.*25*
No income	215 (45.9%)	196 (45.1%)	*0*.*79*
Had a partner	174 (34.7%)	166 (35.5%)	*0*.*79*
Unstable housing†	67 (14.2%)	71 (15.9%)	*0*.*46*
Lived:			*0*.*72*
Alone	74 (14.8%)	60 (12.8%)	
With parents	203 (40.5%)	205 (43.6%)	
With a partner (as a couple)	113 (22.6%)	105 (22.3%)	
Other	111 (22.2%)	100 (21.3%)	* *

Data are mean (SD) or n (%).

*Difficulty reading French or writing in French were self-reported and assessed by the question “In daily living, do you read/write in French with great, some or no difficulty?”.

†Being unstably housed was defined as being hosted by friends or relatives or being a squatter.

With regard to the primary outcome, 63.3% of the participants in the intervention group participated in a training session compared to 55.6% in the control group (p = 0.039), an absolute difference of 7.7 points (95% CI 0.4 to 14.9) (Table 2). There was a significantly higher training session participation rate in the intervention group than in the control group for the women (66.3% *vs* 51.1%; p = 0.003), the younger participants (65.3% *vs* 54.9%; p = 0.041), those unstably housed (79.4% *vs* 52.3%; p = 0.013) and/or who had not continued their education beyond middle school (85.7% *vs* 45.2%; p = 0.003) or high school (62.7% *vs* 53.4%; p = 0.045) (Tables 3 and 4). However, the training session participation rate was the same in each group, regardless of the participants’ health status reported at baseline (179, or 62.8%, in the intervention group *vs* 151, or 55.7%, in the control group [p = 0.09] for the participants in very good and good health and 46, or 64.8%, *vs* 37, or 55.2%, respectively [p = 0.251] for the others). In per-protocol analysis, 163, or 63.9%, of the participants in the intervention group who actually visited a doctor participated in a training session during the year following study inclusion versus 188, or 55.6%, of those in the control group (p = 0.023).

**Table 2:** Outcomes in the intervention and control groups: pre-intervention, intent-to-treat analysis and observed differences.

	Pre-intervention			Post-intervention: Intent-to-treat analysis			Difference in the proportion of favourable changes between the intervention and control groups	
	Intervention Group (n = 504)	Control Group (n = 472)	*p*	Intervention Group (n = 363)	Control Group (n = 341)	*p*	Difference (95% CI)	*p*
**Met outcome measure during the year following the intervention**								
Participated in a training session (primary outcome)				226 (63.3%)	188 (55.6%)	***0*.*039***		
Worked				281 (78.3%)	259 (76.4%)	*0*.*56*		
**Overall health**								
Good or very good	405 (80.7%)	385 (81.6%)	*0*.*72*	284 (83.5%)	297 (82.3%)	*0*.*66*	1.1 (-3.6 to 5.7)	*0*.*64*
**Use of care**								
Had medical insurance	445 (89.0%)	432 (91.7%)	*0*.*15*	336 (93.1%)	321 (94.7%)	*0*.*37*	1.5 (-2.7 to 5.6)	*0*.*47*
Had supplemental health insurance	360 (72.1%)	331 (70.4%)	*0*.*55*	307 (85.5%)	281 (82.9%)	*0*.*34*	-0.4 (-6.2 to 5.4)	*0*.*89*
Had a regular GP	339 (67.5%)	347 (73.5%)	***0*.*040***	310 (85.9%)	273 (80.8%)	*0*.*07*	6.6 (0.9 to 12.2)	***0*.*023***
Had seen a psychologist during the previous year	46 (9.1%)	38 (8.1%)	*0*.*55*	61 (16.8%)	30 (8.8%)	***0*.*002***	4.5 (0.2 to 8.9)	***0*.*039***
Had foregone care during the previous year	155 (31.1%)	127 (27.0%)	*0*.*15*	92 (25.8%)	88 (26.0%)	*0*.*94*	2.3 (-3.0 to 7.6)	*0*.*40*
**Health knowledge**								
Knew that the morning-after pill is taken the day after missing a birth control pill				196 (53.3%)	156 (45.9%)	***0*.*026***		
Knew that the morning-after pill does not protect against AIDS or STIs				319 (88.6%)	300 (88.2%)	*0*.*88*		
**Health behaviours**								
Was doing something to maintain his/her health	233 (46.4%)	206 (43.8%)	*0*.*42*	185 (51.1%)	165 (48.5%)	*0*.*50*	-3.4 (-9.2 to 2.3)	*0*.*24*
Through diet	55 (24.7%)	53 (27.2%)	*0*.*58*	51 (28.2%)	45 (28.0%)	*0*.*91*	5.6 (-3.0 to 14.0)	*0*.*20*
Through sports	71 (77.0%)	152 (77.8%)	*0*.*82*	149 (82.3%)	122 (74.9%)	*0*.*09*	-3.4 (-13.5 to 6.8)	*0*.*52*
No contraception during last intercourse				68 (19.1%)	83 (25.1%)	*0*.*06*		

Data are n (%), unless indicated otherwise.

**Table 3:** Comparison of post-outcome measures between the intervention and control groups by subgroups (gender and age).

	Gender						Age					
	Male			Female			≤ 21 years			> 21 years		
	Intervention Group (n = 182)	Control Group (n = 149)	*p*	Intervention Group (n = 181)	Control Group (n = 192)	*p*	Intervention Group(n = 194)	Control Group(n = 187)	*p*	Intervention Group (n = 166)	Control Group (n = 152)	*p*
**Met outcome measure during the year following the intervention**												
Participated in a training session	108 (60.3%)	91 (61.5%)	*0*.*83*	118 (66.3%)	97 (51.1%)	***0*.*003***	124 (65.3%)	101 (54.9%)	***0*.*041***	100 (61.0%)	86 (56.6%)	*0*.*43*
Worked	148 (82.2%)	115 (77.7%)	*0*.*31*	133 (74.3%)	144 (75.4%)	*0*.*81*	149 (77.2%)	142 (76.3%)	*0*.*84*	130 (79.3%)	116 (76.8%)	*0*.*60*
**Overall health**												
Good or very good	155 (85.7%)	129 (87.2%)	*0*.*69*	142 (78.9%)	155 (80.7%)	*0*.*66*	157 (80.9%)	154 (82.8%)	*0*.*64*	137 (83.5%)	128 (84.2%)	*0*.*87*
**Use of care**												
Had medical insurance	165 (91.2%)	137 (93.2%)	*0*.*50*	171 (95.0%)	184 (95.8%)	*0*.*70*	179 (92.3%)	174 (93.6%)	*0*.*76*	154 (93.3%)	145 (96.0%)	*0*.*29*
Had supplemental health insurance	148 (81.8%)	116 (78.9%)	*0*.*52*	159 (89.3%)	165 (85.9%)	*0*.*32*	162 (84.4%)	152 (81.7%)	*0*.*49*	142 (86.6%)	127 (84.1%)	*0*.*53*
Had a regular GP	145 (80.1%)	110 (74.3%)	*0*.*21*	165 (91.7%)	163 (85.8%)	*0*.*08*	169 (87.1%)	152 (82.2%)	*0*.*18*	138 (84.2%)	119 (78.8%)	*0*.*22*
Had seen a psychologist during the previous year	32 (17.6%)	10 (6.7%)	***0*.*003***	29 (16.0%)	20 (10.4%)	*0*.*11*	35 (18.0%)	12 (6.4%)	***0*.*001***	24 (14.5%)	18 (11.8%)	*0*.*49*
Had foregone care during the previous year	40 (22.5%)	33 (22.3%)	*0*.*97*	52 (29.1%)	55 (29.0%)	*0*.*98*	47 (24.5%)	42 (22.7%)	*0*.*69*	45 (27.8%)	46 (30.5%)	*0*.*60*
**Health knowledge**												
Knew that the morning-after pill is taken the day after missing a birth control pill	88 (48.6%)	65 (43.9%)	*0*.*40*	108 (60.0%)	91 (47.4%)	***0*.*015***	102 (52.9%)	72 (38.7%)	***0*.*006***	92 (55.8%)	84 (55.3%)	*0*.*93*
Knew that the morning-after pill does not protect against AIDS or STIs	159 (88.3%)	128 (86.5%)	*0*.*62*	160 (88.9%)	172 (89.6%)	*0*.*83*	165 (85.5%)	161 (86.6%)	*0*.*77*	152 (92.1%)	137 (90.1%)	*0*.*53*
**Health behaviours**												
Was doing something to maintain his/her health	108 (59.3%)	86 (58.1%)	*0*.*82*	77 (42.8%)	79 (41.2%)	*0*.*75*	94 (48.5%)	76 (40.9%)	*0*.*14*	90 (54.6%)	89 (58.6%)	*0*.*47*
Through diet	19 (18.3%)	20 (23.3%)	*0*.*40*	32 (41.6%)	25 (33.3%)	*0*.*30*	22 (24.2%)	18 (23.7%)	*0*.*94*	28 (31.5%)	27 (31.8%)	*0*.*97*
Through sports	99 (95.2%)	73 (84.9%)	***0*.*016***	50 (64.9%)	49 (65.3%)	*0*.*96*	77 (84.6%)	61 (80.3%)	*0*.*46*	71 (79.8%)	61 (71.8%)	*0*.*22*
No contraception during last intercourse	31 (17.4%)	40 (27.2%)	***0*.*033***	37 (20.8%)	43 (23.4%)	*0*.*55*	36 (19.1%)	39 (21.7%)	*0*.*53*	30 (18.3%)	43 (28.9%)	***0*.*027***

**Table 4:** Comparison of post-outcome measures between the intervention and control groups by subgroups (type of housing and level of education).

	Type of housing						Level of education								
	Unstable			Stable			Middle school			High school			Postsecondary		
	Intervention Group (n = 34)	Control Group (n = 45)	*p*	Intervention Group (n = 305)	Control Group (n = 276)	*p*	Intervention Group (n = 21)	Control Group (n = 32)	*p*	Intervention Group(n = 242)	Control Group(n = 221)	*p*	Intervention Group(n = 98)	Control Group (n = 88)	*p*
**Met outcome measure during the year following the intervention**															
Participated in a training session	27 (79.4%)	23 (52.3%)	***0*.*013***	184 (61.3%)	156 (56.9%)	*0*.*28*	18 (85.7%)	14 (45.2%)	***0*.*003***	148 (62.7%)	117 (53.4%)	***0*.*045***	59 (60.2%)	57 (64.8%)	*0*.*52*
Worked	25 (73.5%)	31 (68.9%)	*0*.*65*	211 (76.7%)	238 (78.8%)	*0*.*55*	16 (76.2%)	17 (53.1%)	*0*.*09*	179 (74.6%)	168 (76.7%)	*0*.*60*	84 (87.5%)	74 (84.1%)	*0*.*51*
**Overall health**															
Good or very good	28 (82.4%)	33 (75.0%)	*0*.*44*	255 (84.2%)	236 (85.5%)	*0*.*65*	12 (57.1%)	27 (84.4%)	***0*.*028***	195 (80.9%)	183 (82.8%)	*0*.*60*	88 (90.7%)	74 (85.1%)	*0*.*24*
**Use of care**															
Had medical insurance	32 (94.1%)	41 (93.2%)	*0*.*87*	287 (94.4%)	262 (95.3%)	*0*.*64*	19 (90.5%)	27 (87.1%)	*0*.*71*	223 (92.9%)	208 (94.1%)	*0*.*60*	92 (93.9%)	86 (98.9%)	*0*.*08*
Had supplemental health insurance	28 (82.4%)	33 (75.0%)	*0*.*44*	269 (88.8%)	235 (85.1%)	*0*.*19*	15 (71.4%)	26 (81.3%)	*0*.*40*	206 (85.8%)	180 (81.8%)	*0*.*24*	11 (11.5%)	12 (13.8%)	*0*.*63*
Had a regular GP	25 (75.8%)	30 (68.2%)	*0*.*47*	267 (87.8%)	230 (93.9%)	*0*.*18*	17 (85.0%)	26 (81.3%)	*0*.*73*	203 (84.2%)	175 (79.9%)	*0*.*23*	89 (90.8%)	72 (82.3%)	*0*.*10*
Had seen a psychologist during the previous year	5 (14.7%)	8 (17.8%)	*0*.*72*	50 (16.4%)	19 (6.9%)	***<0*.*001***	3 (14.3%)	2 (6.3%)	*0*.*33*	37 (15.3%)	18 (8.1%)	***0*.*018***	21 (21.4%)	10 (11.4%)	*0*.*07*
Had foregone care during the previous year	7 (20.6%)	12 (27.9%)	*0*.*46*	76 (25.4%)	71 (25.8%)	*0*.*91*	5 (23.8%)	7 (21.9%)	*0*.*87*	61 (25.5%)	50 (22.7%)	*0*.*49*	24 (25.3%)	31 (36.1%)	*0*.*12*
**Health knowledge**															
Knew that the morning-after pill is taken the day after missing a birth control pill	17 (50.0%)	19 (43.2%)	*0*.*55*	170 (55.9%)	123 (44.6%)	***0*.*006***	10 (47.6%)	15 (46.9%)	*0*.*96*	126 (52.5%)	89 (40.3%)	***0*.*009***	59 (60.2%)	52 (59.8%)	*0*.*95*
Knew that the morning-after pill does not protect against AIDS or STIs	29 (87.9%)	37 (84.1%)	*0*.*64*	269 (88.5%)	244 (88.4%)	*0*.*98*	18 (85.7%)	26 (81.3%)	*0*.*67*	207 (86.6%)	194 (87.8%)	*0*.*71*	92 (93.9%)	80 (92.0%)	*0*.*61*
**Health behaviours**															
Was doing something to maintain his/her health	17 (50.0%)	20 (45.5%)	*0*.*69*	154 (50.7%)	137 (49.6%)	*0*.*81*	7 (33.3%)	10 (31.3%)	*0*.*87*	117 (48.6%)	106 (48.0%)	*0*.*90*	60 (61.2%)	49 (56.3%)	*0*.*50*
Through diet	4 (23.5%)	8 (44.4%)	*0*.*19*	44 (29.0%)	34 (25.2%)	*0*.*48*	1 (14.3%)	1 (10.0%)	*0*.*79*	36 (31.9%)	26 (24.8%)	*0*.*25*	14 (23.3%)	18 (39.1%)	*0*.*08*
Through sports	15 (88.2%)	12 (66.7%)	*0*.*13*	122 (80.3%)	104 (77.0%)	*0*.*51*	5 (71.4%)	7 (70.0%)	*0*.*95*	92 (81.4%)	80 (76.2%)	*0*.*35*	51 (85.0%)	35 (76.1%)	*0*.*25*
No contraception during last intercourse	12 (36.4%)	19 (45.2%)	*0*.*44*	53 (17.8%)	58 (21.6%)	*0*.*24*	3 (14.3%)	12 (40.0%)	***0*.*047***	54 (22.9%)	53 (24.7%)	*0*.*66*	10 (10.3%)	18 (20.9%)	***0*.*046***

Data are n (%).

As for the secondary outcomes, no significant difference was observed between the intervention group and the control group in terms of the employment rate during the year following the intervention or in terms of perceived overall health. Similarly, the intervention did not seem to have any effect on social security coverage or on foregoing care, but significantly more intervention group participants saw a psychologist during the year following the intervention (16.8% *vs* 8.8%; p = 0.002), especially among the men, those in a stable housing situation, and those who had not continued their education beyond high school (Tables 3 and 4). In addition, of those in the intervention group who visited a psychologist, 37 (60.7%) did so at least twice. Of the participants who had not seen a psychologist during the year preceding study inclusion, 11.6% of those in the intervention group *vs* 7.0% of those in the control group (p = 0.039) consulted one during the 12-month follow-up, a 4.5-point difference in the proportion of favourable changes between the two groups. Similarly, of those who had not had a regular GP at baseline, 21.1% now had one at 12 months in the intervention group *vs* 14.5% in the control group (p = 0.023), a 6.6-point difference in the proportion of favourable changes between the two groups.

On the subject of health knowledge, a higher proportion of participants in the intervention group knew when to use the morning-after pill than in the control group (53.3% *vs* 45.9%; p = 0.026). This difference was significant among the women but not among the men, among the participants under the age of 21 years, and those who were stably housed and/or who had not continued their education beyond high school.

Overall, in both groups, a higher proportion of men than women engaged in sports to maintain their health (253 or 90.0%, *vs* 151, or 66.2%; p<0.001). Moreover, a higher proportion of men in the intervention group engaged in sports to maintain their health than in the control group (95.2% *vs* 84.9%; p = 0.016). A significantly smaller proportion of men in the intervention group did not use a method of contraception during their last sexual intercourse than in the control group (17.4% *vs* 27.2%; p = 0.033), while for the women, no significant difference was observed between the two groups for these two variables. In addition, a higher proportion of the older participants in the intervention group used a means of contraception during their last intercourse than those in the control group.

In the intervention group, the participants who had actually attended a social and preventive medicine consultation and those who had not were broadly comparable in terms of age, gender, the level of education, difficulty reading French, income, having or not having a partner, the type of housing, household composition and baseline overall health. On the other hand, in the intervention group, a higher proportion of those who ended up attending a social and preventive medicine consultation were French, born to two French parents or foreign immigrants (respectively, 215, or 65.8%, *vs* 102, or 57.6%, and 34, or 10.4% *vs* 13, or 7.3%; p = 0.023), and had difficulty writing in French (104, or 31.8%, *vs* 40, or 22.6%; p = 0.029) than of those who had not attended a consultation.

There were no significant effects on the primary outcome based on the ML, nor on the type of the ML (urban MLs *vs* medium-sized towns MLs).

No unintended effects were reported in either group.

## Discussion

This study showed how systematically offering a social and preventive medicine consultation could improve training participation among the NEETs who visited MLs in France. This effect was more pronounced in women, participants under 21 years of age, those unstably housed and those who stopped their education earlier. There was no evidence of a differential effect according to perceived overall health at baseline.

Reducing the unemployment rate among young people has been and still is a priority in developed countries, both for societal and economic reasons, but also, of course, for improving the well-being, social integration and health of the young people concerned. This study was neither designed nor powered to detect employment but instead used an intermediate outcome (participation in training sessions). As expected, no significant difference in terms of returning to the labour market was observed during the year following study inclusion (a very short period of time for such an event), but the aim of training among NEETs is to enhance their employability by giving them the support and skills needed to make a successful transition to the job market. It has been shown that the return to the labour market in the short and long term is better for people who have access to training [21,22].

In their meta-analysis of re-engagement interventions among NEETs, Mawn and colleagues found that high-intensity multicomponent interventions featuring classroom and job-based training appear to increase employment amongst NEETs by 4% compared to controls. This is especially true when jobseekers are young [23]. In our study as well, the intervention worked better among younger participants. Even though a single meeting with either a social worker or a physician is a very low contact rate (and therefore the ‘intervention dose’ is very low), this study provides some preliminary evidence that multi-component interventions might work better than one-field intervention. Also, we think our intervention may have worked better because this single meeting might have made it more likely for participants in the intervention group to show up at a training session.

NEETs constitute an exceedingly heterogeneous population. It comprises several subgroups, each having its own characteristics and needs. That said, it is important to identify the characteristics and needs of the different subgroups that require specific forms of policy intervention, such as in the form of welfare or providing training [1]. This diversity among NEETs was encountered in our study population, as was the diversity of the effects of the intervention evaluated in different subgroups. It is interesting to note that this intervention was effective in those whose prevention and health needs were certainly greater, such as young women, the participants who were unstably housed and those who were less educated. In general, interventions among NEETs are less effective with those most disadvantaged, and there was, similarly, reason to fear that our primary care intervention would obey the inverse care law [21,24].

Regarding its primary purpose, our intervention had an impact on the women but not on the men, similar to the "Jovenes en Accion” intervention carried out in Colombia in 2005, although no hypothesis was offered in an attempt to explain this difference [25]. The authors of the 2017 meta-analysis hypothesized that “females had benefitted more from the intervention because of their possible lower levels of labour market engagement relative to males in control populations” [21]. The employment rate in our control group was, in fact, lower among the women than the men. More generally, it is known that in France, since 2009, in the most disadvantaged neighbourhoods and at a time when there is continually increasing unemployment, working-age women have been gradually withdrawing from the labour market: 50% of them are no longer in the job market compared to 30% of men, and they hold part-time jobs three to four times more often than men, (in more than half of the cases) [26].

We acknowledge certain limitations in both the design and conduct of this study. We chose to present in this paper the comparison between the intervention group and the control group but not that between the social worker group and the control group. This was done for two reasons. One was that the effect of solely the intervention by the social worker alone was nil (results not shown). The other was that, like other authors, we assumed that a multi-component intervention would be more effective [21]. In the end, the study population was smaller than anticipated because of a higher-than-expected lost to follow-up rate (although it was the same in both groups). However, we nonetheless observed our intervention to have a significant effect, with a power of 70%. The lost to follow-up rate can be explained, among other things, by the difficulties that the young people had in getting to the MLs to take the final questionnaire (busy with training, a work term, working, transportation problems, etc.). We do not have any information on the fate of these participants lost to follow-up, but, broadly speaking, they were more vulnerable than those who were followed. It is therefore possible that self-selection operated among the most vulnerable NEETs whereby the most motivated ones were retained in the study (and at the MLs?) and which could also explain, at least in part, why the intervention was more effective in these followed vulnerable participants than in the others who were followed. Lastly, in the baseline questionnaires and at 12 months, certain information was missing, information that would have been useful for better describing this population and understanding the reasons for the intervention’s success or failure, specifically, more-detailed information on their work history and health-related information (e.g. smoking status, drug use, etc.). It had been decided–for ethical and data quality reasons–to have this information collected by the physicians. It is therefore available only for the young people in the per-protocol intervention group. Yet, only 65% of the young people in the intervention group actually attended a social and preventive medicine consultation. They were broadly comparable to those who did not attend for most of the study variables, including overall health at baseline, except for migration origin and the ability to write in French (probable connection). This proportion can be considered a success for young people whose young age, socially disadvantaged situation and frequent isolation from their families are not conducive to the use of health-care services. Conversely, a third of the young people did not want this service systematically offered by the research assistants. Strategies would no doubt be needed on a routine basis to improve their motivation, such as information and reminders from the personal counsellor, information and advertising on the MLs’ premises, and young people sharing their experiences with one another. Lastly, if this intervention is generalized to all the MLs, this would probably require greater formalization of the contents of the consultations offered. The physicians who participated in this experiment actually had lengthy experience with this type of consultation and the target population. It is not certain that all MLs would have access to such experienced primary care physicians.

Despite its limitations, this study is the first of its kind in France and, to our knowledge, the first of its kind to be published in the scientific literature. Indeed, no randomised controlled trial had ever been conducted to examine the effect of a health intervention on the training participation rate among NEETs (or on their return to the labour market). As well, there has been little research to examine NEETs’ physical and mental health outcomes. This is surprising, given the well-established bidirectional relationships between unemployment and health. Yet it is more than likely that the barriers to accessing preventive and social medicine consultations that are described in the general population are even greater in this population. In primary care, it is unfortunate that such primary care practices–focused on health promotion and disease prevention–are not widely practiced and accessible [27,28]. Different reasons can explain this at three different levels: at the physician level (e.g., uncertainty due to conflicting recommendations, a lack of time, and a lack of knowledge of young people’s needs and expectations), at the patient level (e.g., fear of consulting a doctor, unawareness of services and their benefits, a lack of autonomy, competition with other basic needs), and, lastly, at the health-care system level (e.g., a lack of low-threshold services and/or services integrated into the social services dedicated to these underserved, hard-to-reach populations) [29]. For instance, in France, preventive medical consultations are available (under conditions provided by law or the welfare system) at schools, universities, in the workplace, and for poor persons over 25 years of age. This means that NEETs are falling between the cracks in terms of the existing dedicated preventive medical services. In our study, 38% of the intervention group participants reported afterwards that they needed this consultation, even though they had not previously thought that they did, and 60% found that they learned things about health during the consultation. After this single consultation, certain changes in health behaviours were observed. The men in the intervention group used contraception more often than those in the control group, and a higher proportion of them reported, at 12 months, engaging in sports to improve their health than in the control group. As for the women, a higher proportion of them now knew when and in what circumstances to use the morning-after pill. As well, a higher proportion of the participants in the intervention group who did not have a regular GP now had one than in the control group. Another positive result was that a higher proportion of the young people saw a psychologist after the intervention (and began a follow-up with him/her). This is especially important because mental health in this population is particularly poor and because it is a known fact that the better a young person’s mental health and self-esteem, the better their chances of finding a job [10,30,31].

It is very difficult to demonstrate the effectiveness of intervention studies involving primary care prevention. Indeed, it is not easy to document that changes in risk factors or disease prevalence can be achieved by means of preventive service programs or that reducing risk factors or disease leads to less morbidity and mortality (in a manner of speaking, preventive success is a nonevent) [29]. From this standpoint, having chosen an intermediate indicator that was observable in the short term was one of the key factors for this trial’s success. We find these positive results especially important because once the value of preventive services is recognized and supported, programs and systems for implementing them can be introduced and funded. In the Paris region, for instance, the results of our study have convinced the decision-makers at the regional health agency (*Agence régionale de santé d’Ile-de-France*) to put improvements in NEETs’ health at the top of the priorities for the 2013–2017 Regional Programme for Access to Prevention and Primary Care (*Programme Régional d’Accès à la Prévention et aux Soins*). For future trial recommendation, we would suggest to follow up the participants at 6 months (and one year). Indeed, this population is in transition and it has been demonstrated that the quicker the intervention is after receiving the status of NEET (to help young people to find a job or to enroll in the regular education system), the better his chances are in the job market, reducing ‘scarring’ and ‘wage penalties’ [1].

As reported in 2016 by the Lancet Commission on Adolescent Health and Wellbeing, in health promotion and prevention, just as in marketing, interventions that influence attitudes, values, and behaviours are likely to be more impactful during adolescence or early adulthood than at any other period of life [32–34]. Many of the benefits conferred by preventive medicine are achieved only after a long period of time and consequently require an investment in the future. However, early adulthood is characterized by the end of a period of high brain plasticity associated with adolescence in which the final phase of adult brain organization takes place [35]. As well, during adolescence and young adulthood, people acquire the physical, cognitive, emotional, social, and economic resources that constitute the foundation for their health and wellbeing later in life [36]. These very resources establish trajectories into the next generation. Investing in the health and wellbeing of adolescents and young adults–especially those, such as NEETS, at greatest need–is providing benefits now and will do so in the coming decades and for the next generation.

## Supporting information

S1 Appendix CONSORT 2010 checklist of information to include when reporting a randomised trial. (DOC)

S2 Appendix Trial study protocol. (DOCX)

S1 Table Baseline characteristics of follow-up and lost to follow-up participants among the 976 patients randomised to the intervention and control groups. (DOCX)

S2 Table Comparison of the lost to follow up participants’ baseline characteristics by group. (DOCX)
