# Supplementary material for: Effects of a systematically offered social and preventive medicine consultation on training and health attitudes of young people not in employment, education or training (NEETs): An interventional study in France
Source: PLoS One. 2019 Apr 26;14(4):e0216226. doi: 10.1371/journal.pone.0216226 (PMC6485762; doi:10.1371/journal.pone.0216226)
Supplement: S2 Appendix — (DOCX) [file pone.0216226.s002.docx]

**Projet de recherche sur la santé des jeunes (PResaJe)**

**Protocol**

**Scientific responsible** : Pierre Chauvin, MD, PhD

Directeur de recherche Inserm

Equipe DS3

INSERM & Paris VI – UMRS 707

27 rue de Chaligny, 75571 Paris Cedex 12, France

Email : [pierre.chauvin@upmc.fr](mailto:pierre.chauvin@upmc.fr)

1. **Background**

Despite very advantageous health care and insurance systems, there are still major discrepancies in health status among the population. Several studies show that these disparities are correlated with socioeconomic status (Wilkinson, 1997). Poor people have higher risks of disease, morbidity (Arber, 1997) and mortality (Pappas, 1993) than the wealthier persons. This empirical result was also found for adolescents and young adults (Power, 1991). In a comparative study focusing on Britain and Finland, Rahkonen et al. (1995) find that social class differences and the level of education are two main explanatory factors of differences in health among young adults. Moreover, disparities in childhood socioeconomic circumstances have an effect on adult health and future health-related behaviors (Van de Mheen, 1998 ; Melchior, 2007 ; Poulton, 2002).

Many health professionals sustain the idea that inequalities in health access are one of the main factor explaining discrepancies in health status (Andrulis, 1998). Difficulties in financing care represents a real threat to health and well-being for persons who live in poverty. Using the fact that uninsured low-income adults in Oregon were randomly chosen to apply for Medicaid, Finkelstein et al. show that a better access to public health insurance lead to a higher health care utilization, low out-of-pocket expenditures and better health outcomes. Currie and Gruber (1996) find the same results for low-income children showing that public insurance significantly increase the use of medical care and improve child health by reducing mortality.

However, others argue that eliminating financial barriers to health care is not an efficient solution to reduce disparities in health status. In a study conducted in Canada, Dunlop et al. (2000) show that people less educated and with lower income use physicians' services at a lower rate that those with higher income and level of schooling despite the existence of an universal health care system. Health insurance coverage is not sufficient to reduce disparities in health between socioeconomic groups (Adler et al., 1993). In the RAND health insurance experiment, Manning et al. (1987) do not find effects from a better insurance coverage on health outcomes. Universal coverage do not succeed in eliminating socioeconomic disparities in health status.

This study, conducted in five Missions locales, aims at testing the efficiency of two programs. Both interventions target young unskilled people from 18 to 25 years old. The first intervention aims to optimize health coverage in order to reduce or eliminate financial barriers to health access. A social worker is in charge of finding the most advantageous coverage, assisting young people during their administrative processing and providing information on the French public health and private insurance systems. The second treatment consists on a double intervention. The first part is the same as the one described as the first intervention. But it is completed with medical consultations. Indeed, participants randomized in this group are encouraged to visit the health professionals of the structure. During these visits, GP's and psychologists are in charge of providing information on the health care system and health, doing a global health check-up and sending participants to a specialist if need be.

This randomized experiment aims at measuring the impact of the two interventions over a 12-month period. It focuses on the impact on health demand and access but also on social integration and employment.

1. **Outcomes**
   1. **. Primary outcome**

To assess impact of health issue and its screening on professional training and social autonomy of unemployed youth.

- 1. **. Secondary outcomes**

Description of health outcomes and prevention knowledge of this population aged 18 to 25 years old

To improve the content and the frequency of social and preventive medical consultations

1. **Design**
   1. **Type of study**

It will be a parallel unmasked randomised parallel interventional study.

- 1. **Participants**

***Participant identification***

Participants will be recruited among Missions locales.

5 Missions locales have been selected : Toulouse, Sénart, Clichy, Reims, Poitiers. These structures have been selected according to their interest for the study and their capacities of implementing the empirical design. Because one treatment consists of the intervention of health professionals, only *Missions locales* with a GP can take part in the study.

Any young people coming for the second time in the selected Missions Locales will be targeted in this study. Because more than a half of young people visit only one time the structure, we will select only those who will from a long-term counseling in order to reduce the attrition bias.

After finishing their meeting with their counselor, young people will go to the member of the field staff who will present the study design.

The randomization and inclusion in the study will run during one year. Each participant will be followed up during one year after completing the baseline questionnaire.

***Exclusion criteria***

Participants will be ineligible if they are unable to speak or to understand French and consequently to answer to the questionnaire (without a translator). As said before, people are included in the study after their second visit in the *Missions locales*.

People who have a serious disability that leads to special accomodations for work are not eligible in the study. Also, people who have an acute disease that lead them to have an emergency intervention will not be eligible.

***Stopping rule and lost to follow-up***

No stopping rule is planned. All the recruited participants will be included for analyses.

***Sample size***

We target a sample of 1 443 participants randomized in three groups. It means 481 participants in each group. Sample size calculations are based on the ability to detect an impact of the treatment in our outcomes.

About 55% percent of the users of the Missions locales go to a training session. To detect an increase of 10 points of each intervention with the control group (one-sided test), we need to have 409 patients in each group with a 90% power and an alpha risk of 0.05. We estimate than 15% of participants will be lost-to-follow-up, so it’s necessary to include at least 481 participants in each group.

***Randomization***

All participants are randomly allocated to one of the three groups: the first treatment group (1), the second treatment group (2) and the control group (3). Research assistants are in charge of doing the allocation to study groups by entering names and birth dates of participants in an algorithm which attributes randomly a number of cohorts (1, 2 or 3) for each participant.

Randomization occurs on receipt and signature of informed consent and baseline questionnaire. People can refuse to participate. This decision doesn’t influence the modality of counseling offered by the *Missions locales*.

***Planned timetable***

Starting date for the recruitment: november 2010

Ending date for the recruitment: november 2011

Starting date of the follow-up: november 2011

Ending date of the follow-up: novembre 2012

***Statistical analyses***

Statistical analyses will be done on Epi-Info^®^, Statview^®^ , Stata^®^, or SAS^®^. All the analyses will be transferred to the steering comitee of the study.

- 1. **Intervention**

***First intervention***

Participants randomized in the first treatment group are encouraged to meet a social worker. The main objective of this treatment is to reduce or eliminate financial barriers to health access. Taken into account the initial situation of participants regarding to the health insurance system, the social worker is first in charge of finding the most advantageous coverage among the different types of *regimes*. The social worker also assists young people in all their administrative processing with the Social Security by collecting documents necessary for subscribing for a public health coverage. He can also propose to subscribe to a private insurance or *mutuelles* for the last third which leave a co-payment not reimbursed by the public insurance. Finally, the last part of this intervention consists of providing basic knowledge on the French health care and insurance systems.

***Second intervention***

Participants randomized in the second treatment group benefit from the first intervention described above but, in addition, they are also encouraged to visit health professionals working in the *Missions locales*.

These consultations with the GP aim first at investigating the young's health status, his level of health services' use and sending him to specialist in case of need. If the first appointment is systematic, participants are free to decide the frequency of the next consultations. The main goal of this second treatment is to encourage health-related behaviors among young adults by giving useful advices, increase comfort with the health care system and their belief in its capacity to satisfy their expectations.

***Control group***

Participants in the control group benefit from the normal program offered by the *Mission locale* as both experimental groups. They are not encouraged to meet health professionals or social workers. But in case of need, they can take an appointment with health professionals.

- 1. **Recruitment and training of field staff**

***Research assistants***

Five research assistants, one for each study site, will be recruited and trained during several sessions. The first sessions aim at presenting the study protocol. Indeed, research assistants are in charge of implementing the protocol in their site. They will also be trained on the baseline and the 12-months questionnaire. Questionnaires will be administered during a face-to-face interview with the participants. Research assistants have to be trained on the way of conducting a survey.

***Social workers***

Three part-time social workers will be also recruited, one for the *Missions locales* located in Toulouse, Reims and Poitiers. For the two remaining *Missions locales* (Clichy and Sénart), the research assistants will assume the functions of social worker. In fact, these two *Missions locales* are smaller and receive less young people than the three others. Research assistants will have enough time to assume the two missions. Social workers will be trained during one session. The main goal of this training will be to harmonize interventions conducted in the five experimental sites. The study involves homogenizing practices and the content of the provided information. The training aims also at defining a common way of coding the pre- and after-treatment health coverage status of each participant in order.

***Health professionals***

The second treatment implies the interventions of health professionals. In order to reduce the cost of interventions, we select only *Missions locales* which already employ doctors and psychologists. Psychologists accept to participate in two sites (Toulouse and Sénart). A meeting with GP’s will be organized. Because each GP has his one practice of medicine, the first objective, as for social workers, will be to harmonize the content of their interventions. GP’s will also be in charge of a part of the data collection by filling in a medical questionnaire. In fact, some information about participants’ health status will be directly collected by health professionals during the consultation. Doctors will be trained on the medical questionnaire.

***Pilot phase***

A pilot phase will take place during three months. During this pilot, the field staff will be trained. Several training sessions for the different actors of the project will be organized by the study team.

The protocol will be tested in each experimental site and adapted to fit with the specificities of the five *Missions locales*. Indeed, each *Mission locale* has its own organization. The main difficulty will be to implement a harmonized protocol in spite of particularities of the five experimental sites. Some adjustments will be needed.

A phase of coordination will be also necessary to ensure the support of members of the *Missions locales*’ staffs. They will have to be informed on the objectives of the study, the design and the methods used by the project team. We will ensure that they will not interfere with the study. The pilot will give also the possibility to test baseline and medical questionnaires.

1. **Outcomes measures**
   1. **Baseline questionnaire**

This survey instrument is designed by the study team by adapting different modules from existing survey. Different versions of this baseline survey will be tested by research assistants on young people visiting the *Missions locales* during the pilot phase. Data will be collected pre intervention.

**Outcomes**

***Professional and familial situations***

Some questions are asked to collect information about the professional and familial situations of participants. We measure the level of schooling (Q. 6), diplomas obtained (Q. 7), the potential reasons of dropping out (Q. 8, 9 and 10), the adequacy of the professional formation to expectations (Q. 11) and professional projects (Q. 12). The question 19 asks about resources. The financial situation is also measured by asking the perception of the participant about his own financial situation (Q. 26), if he goes away for vacation during the last year (Q. 27) and if his household (Q. 28) or he has debt or credits (Q. 29).

The familial situation is also investigated by asking the marital status (Q. 15) and the composition of the household (Q. 16, Q.17). The question 18 asks the professional situation of the head of household. A special module (F) asks questions about the profession, nationality and the nature of relationship with both parents. It aims also at collecting information about childhood by questioning who was in charge of educating the participant (F. 10, 11, 12, 13), where the participant spent his childhood (F. 14, 15, 16) and if he lived difficult episodes during childhood and adolescence. The participant’s feeling about his own childhood and adolescence is also measured (F. 19, 20).

***Social environment***

A module (L) of the baseline questionnaire aims at describing the conditions of housing. Questions about the type of housing (L. 2), the rent (L. 3) and the perception of the accommodations (L. 5, L. 6) are asked.

The neighborhood is also investigated by the questionnaire. The main goal is to measure the reputation and perception of the living area (Q. 1 to Q. 7).

A short module (A) aims at collecting information on the level of social integration by asking participants about activities they practice or associations they are member of. This module is completed by a second one (R) about relations with peers which aims at giving an idea about the level of social integration of the participant. The number of friends (R. 1), the frequency of contacts (R. 2), the feeling about social integration (R. 5, 7) and the type of discrimination (R. 9, 10) the participant suffers from are measured. We try also to measure trust in institutions (R. 8).

***Self-reported health***

A part of our questionnaire aims at measuring self-reported health. Three initial questions are asked to know how the participant perceives his general health status (S. 1), his physical status (S. 2) and his psychological status (S. 3) in a scale with five possibilities (very good, good, medium, bad or very bad). The questionnaire asks if participants suffer from a chronic illness (S. 4), if it constitutes an obstacle in their everyday life (S. 5) and how participants perceive their own health in comparison to others (S. 7).

Three questions focus on weight by asking how others judge the participant’s weight (S. 28), how the participant perceives his own weight (S. 29) and if he would lose or win weight (S. 30). The possible financial strain for buying food is also measured (S. 27).

***Health care use***

Our measure of health care use is based on several questions. The first questions aim at measuring if participants have a regular doctor (S. 10, 11) and if they trust him (S. 12, 13). We examine whether participants use the public hospitals for non-emergency care (S. 14) and for which reasons (S. 15). We ask whether participants visit a GP during the last year (S. 16), others health professionals (S. 17) and specialists (S. 18).

***Access to care***

To assess whether participants have access to care, we ask first if the participant needed to give up medical care during the last year (S. 19) and if it is the case, for which reasons (S. 20) and for which care (S. 21). Two questions focus on dental care (S. 23, 24).

We include in these categories of outcomes questions about the type of health coverage (AD. 5, 6, 7 8.). However, we know that responses to this type of questions could be biased because of the lack of knowledge on the insurance system and the different types of Social Security *regimes*.

***Health knowledge***

Finally, the questionnaire aims at measuring the level of knowledge about three items: consequences of drugs consumption, sexuality and the health insurance system.

- 1. **Medical questionnaire**

This questionnaire is designed by health professionals among the study team. GP’s will fill in this survey during their consultations with participants. We design a medical questionnaire for two main reasons. Doctors are more qualified to collect objective information about health status. Moreover, for ethics questions, some very specific questions about health or health behaviors need to be asked by health professionals who are able to answer to health demand.

- 1. **Endline questionnaire**

***Professional and familial situations***

Some questions are asked to collect information about the professional and familial situations of participants. The familial situation is investigated by asking the composition of the household (Q. 1, Q.2). The question 3 asks the professional situation of the head of household. The question 4 asks about resources.

We measure professional projects (Q. 5) and the evolution of the chance to find a job soon compared to last year. The question 9 asks if the participant have worked in the last year, when was it, was it a full or a part-time job, how many weeks in total, with which kind of contracts (Q. 10-16). Questions 18 to 20 ak if the participants has participated to training sessions, which kind, how many sessions, and how many days. The question 21 asks if the participant has seen the working counsellor.

***Social environment***

Questions R1 to R6 aims at collecting information on the level of social integration by asking participants about activities they practice or associations they are member of. The number of friends (R. 7), the frequency of contacts (R. 8), the feeling about social integration (R. 9-13). We try also to measure trust in institutions (R. 14).

***Self-reported health***

A part of our questionnaire aims at measuring self-reported health. Three initial questions are asked to know how the participant perceives his general health status (S. 1), his physical status (S. 2) and his psychological status (S. 3) in a scale with five possibilities (very good, good, medium, bad or very bad). The questionnaire asks if participants suffer from a chronic illness (S. 4), opinions about the health care system (S. 5, S. 6) and how participants perceive their own health in comparison to others (S. 7). Two questions focus on what partcipants do in order to maintain their health (S. 8, S. 9).

***Health care use***

Our measure of health care use is based on several questions. The first question aims at measuring if participants have a regular doctor (S. 10) and if they trust him (S. 11, 12). We examine whether participants use the public hospitals for non-emergency care (S. 13) and for which reasons (S. 14). Questions S. 15 and S. 16 ask the participant if he went at least once in the ER and for which reason. We ask whether participants visit a GP during the last year (S. 17), others health professionals (S. 18) and specialists (S. 19), dentists (S. 28)

***Access to care***

To assess whether participants have access to care, we ask first if the participant needed to give up medical care during the last year (S. 24) and if it is the case, for which reasons (S. 25) and for which care (S. 26, S. 27). Two questions focus on dental care (S. 23, 24).

We include in these categories of outcomes questions about the type of health coverage (S31 to S37).

***Health knowledge and information***

The first questions of this section are about health information i.e. interest of the participant in health. information, feeling to be informed about different subjects (alcohol, tobacco, cannabis, birth control, STD, AIDS, pollution, food, vaccines, cancer and depression), where to get the information, to have searched in the last year some information in health (C. 1-C. 4). Then questions investigate about the French health system (C. 5, C. 6, C. 8, C. 9, C. 10).

Question C. 7 asks to the participant to answer if some affirmations about morning after-pill are correct or not. Questions C. 11, C. 12 and C. 13 are about sexual intercourse, type of birth-control used during the last sexual intercourse and the provider of the birth-control.

***Satisfaction of the consultation with the doctor in the ML***

The question of this section aims at investigate the level of satisfaction of the medical consultation, the positive and negative aspects of this consultation.

***Satisfaction of the meeting with the social worker in the ML***

The question of this section aims at investigate the level of satisfaction of the meeting with the social worker, the positive and negative aspects of this meeting.

**Beck hopelessness scale**

To finish, we ask all the items of the hopelessness scale to calculate a score that predicts a suicide risk.

1. Data collection, validation, capture and archivage

Data will be collected in a face-to-face questionnaire by research assistant. In each Mission Locale, a person will be chosen for the study monitoring (recruitment, follow-up, lost-to-follow up, late, …). All the data will be informatically captured on an Excel file. After recruitment, all participants will have a number randomly attributed by an alogrithm. The correspondence between numbers and names of participants will be kept by the research assistant and the doctor on a paper (but not on computers). On computer, the data will be only registered with the number of the participant. All the analyses will present only agregated results. The computers that will be used for the data captures won’t be the sames that are used in Missions Locales and will be locked by a personnal password.

All the questionnaires will be kept under key in the office of Dr Pierre Chauvin (located in the Faculté de médecine Pierre et Marie Curie 27 rue Chaligny, 75012 Paris, France) for 15 years and will be available for the study responsibles and the promotor of the study.

All the scientific publications will have to be authorized by the scientific responsible, Dr Pierre Chauvin.

1. Ethics

This study will be conducted according to the last update version (Edimbourg) de la Déclaration d'Helsinki, the recommandations in ethics and good practice in epidemiology adopted in 1999 by l'Association des Epidémiologistes de Langue Française [ADELF 1998], in its updated version of 2008. This protocol has a favourable advice of CNIL (Commission nationale informatique et libertés -number 1527880).

In each Mission locale, a poster will present the study.The signature of the informed consent will be receipt by the research assistant. This consent will remind the background and objectives of the study, the medical data that will be collected, the study duration, the individual beneficies of the study, and the referent person to apply the right to object or modification.

1. Study organisation

The steering committee will be composed of

- Equipe DS3 represented by Pierre Chauvin and Sophie Lesieur

- Mission locale de Sénart represented by Didier DUGAST, Joel DUTERTRE, Virginie KERGOAT

- le CREST (centre de recherche en économie et statistique) represented by Bruno Crépon Simon Beck and Lucile Romanello.

1. Study partners

Mission Locale de Sénart

Principal investigator : Joël Dutertre, médecin, 69 rue du Faubourg Saint Martin 75010 PARIS, 01.40.40.97.45.

Investigateur associate : Virginie Kergoat, Responsable du service Accompagnement de la Mission Locale de Sénart, immeuble "le Sextant" - 3e étage - 462, rue Benjamin Delessert, 77550 Moissy Cramayel, tel : 01.64.13.40.18

Promoter : Didier Dugast, Directeur de la Mission Locale de Sénart, immeuble "le Sextant" - 3e étage - 462, rue Benjamin Delessert, 77550 Moissy Cramayel, tel : 01.64.13.40.18

INSERM

DS3 team will bring skills in the study methodology. The collaboration might be pursued after the collection data for the statistical treatment.

Expertise épidémiologique et statistique : Pierre Chauvin, DR, Sophie Lesieur, IE, UMRS 707 Inserm & université Pierre ou Marie Curie,faculté de médecine St Antoine, 27 rue Chaligny, Paris 75012, tel : 01 44 73 84 65

Funding

Le fond d’experimentation de la jeunesse - Ministère des sports, de la jeunesse et de la vie associative, 95 avenue de France, 75650 Paris Cedex 13

1. References

Adler, N.E., W.T. Boyce, M.A. Chesney, S. Folkman, and S.L. Syme, **“Socioeconomic inequalities in health,”** JAMA: The Journal of the American Medical Association, 1993, 269 (24), 3140.

Andrulis, D.P., **“Access to care is the centerpiece in the elimination of socioeconomic disparities in health,”** Annals of Internal Medicine, 1998, 129 (5), 412.

Arber, S., **“Comparing inequalities in women’s and men’s health: Britain in the 1990s,”** Social Science & Medicine, 1997, 44 (6), 773–787.

Becker, G.S., **“Front matter, Human Capital: A Theoretical and Empirical Analysis, with Special Reference to Education,”** 1975.

Bellanger, M.M. and P.R. Mossé, **“The search for the Holy Grail: combining decentralized planning and contracting mechanisms in the French health care system,”** Health economics, 2005, 14 (S1), S119–S132.

Bloom, D.E. and D. Canning, **“The health and wealth of nations,”** Science, 2000, 287 (5456), 1207.

Chirikos, T.N. and G. Nestel, **“Further evidence on the economic effects of poor health,”** The Review of Economics and Statistics, 1985, pp. 61–69.

Currie, J. and J. Gruber, **“Health insurance eligibility, utilization of medical care, and child health,”** The Quarterly Journal of Economics, 1996, 111 (2), 431.

De Mheen, H. Van, K. Stronks, CWN Looman, and JP Mackenbach, **“Does childhood socioeconomic status influence adult health through behavioural factors?,”** International Journal of Epidemiology, 1998, 27 (3), 431.

Dunlop, S., P.C. Coyte, and W. McIsaac, **“Socio-economic status and the utilisation of physicians’ services: results from the Canadian National Population Health Survey,”** Social Science & Medicine, 2000, 51 (1), 123–133.

Finkelstein, A., S. Taubman, B. Wright, M. Bernstein, J. Gruber, J.P. Newhouse, H. Allen, and K. Baicker, **“The Oregon Health Insurance Experiment: Evidence from the First Year,”** Technical Report, National Bureau of Economic Research 2011.

Imai, Y., S. Jacobzone, and P. Lenain, **“The changing health system in France,”** OECD Economics Department Working Papers, 2000.

Manning, W.G., J.P. Newhouse, N. Duan, E.B. Keeler, and A. Leibowitz, **“Health insurance and the demand for medical care: evidence from a randomized experiment,”** The American Economic Review, 1987, pp. 251–277.

Melchior, M., T.E. Moffitt, B.J. Milne, R. Poulton, and A. Caspi, **“Why do children from socioeconomically disadvantaged families suffer from poor health when they reach adulthood? A life-course study,”** American journal of epidemiology, 2007, 166 (8), 966.

Mushkin, S.J., **“Health as an Investment,”** The journal of political economy, 1962, 70 (5), 129–157.

Pappas, G., S. Queen, W. Hadden, and G. Fisher, **“The increasing disparity in mortality between socioeconomic groups in the United States, 1960 and 1986,”** New England Journal of Medicine, 1993, 329 (2), 103–109.

Perri, T.J., **“Health status and schooling decisions of young men,”** Economics of Education Review, 1984, 3 (3), 207–213.

Poulton, R., A. Caspi, B.J. Milne, W.M. Thomson, A. Taylor, M.R. Sears, and T.E. Moffitt, **“Association between children’s experience of socioeconomic disadvantage and adult health: a life-course study,”** The Lancet, 2002, 360 (9346), 1640–1645.

Power, C., **“Social and economic background and class inequalities in health among young adults,”** Social science & medicine, 1991, 32 (4), 411–417.

Rahkonen, O., S. Arber, and E. Lahelma, **“Health inequalities in early adulthood: a comparison of young men and women in Britain and Finland,”** Social science & medicine, 1995, 41 (2), 163–171.

Rodwin, V.G., **“The health care system under French national health insurance: lessons for health reform in the United States,”** American Journal of Public Health, 2003, 93 (1), 31.

Schoen, C. and M.M. Doty, **“Inequities in access to medical care in five countries: findings from the 2001 Commonwealth Fund International Health Policy Survey,”** Health Policy, 2004, 67 (3), 309–322.

Wilkinson, R.G., “**Socioeconomic determinants of health: Health inequalities: relative or absolute material standards?,”** British Medical Journal, 1997, 314 (7080), 591.
