# Supplementary material for: Effects of a systematically offered social and preventive medicine consultation on training and health attitudes of young people not in employment, education or training (NEETs): An interventional study in France
Source: PLoS One. 2019 Apr 26;14(4):e0216226. doi: 10.1371/journal.pone.0216226 (PMC6485762; doi:10.1371/journal.pone.0216226)
Supplement: S1 Table — (DOCX) [file pone.0216226.s003.docx]

**S1 Table: Baseline characteristics of follow-up and lost to follow-up participants among the 976 patients randomised to the intervention and control groups**

|  | **Lost to follow-up participants (n=272)** | **Follow-up participants**  **(n=704)** | ***p*** |
| --- | --- | --- | --- |
| Age (years) | 21.1 (2.1) | 21.3 (2.0) | *0.26* |
| Gender |  |  | *0.40* |
| Female | 136 (50.0%) | 373 (53.0%) |  |
| Male | 136 (50.0%) | 331 (47.0%) |  |
| Origin |  |  | *0.26* |
| French, born to two French parents | 164 (60.3%) | 464 (65.9%) |  |
| French, born to foreign parent(s) | 81 (9.8%) | 178 (25.3%) |  |
| Foreigner | 27 (9.9%) | 62 (8.8%) |  |
| Level of education |  |  | ***0.020*** |
| Middle school | 35 (12.9%) | 53 (7.6%) |  |
| High school | 178 (65.4%) | 463 (66.0%) |  |
| Postsecondary | 59 (21.7%) | 186 (26.5%) |  |
| Difficulty reading French | 41 (15.1%) | 115 (16.4%) | *0.61* |
| Difficulty writing in French | 87 (32.0%) | 208 (29.6%) | *0.46* |
| No income | 121 (48.2%) | 290 (44.5%) | *0.31* |
| Had a partner | 87 (32.3%) | 253 (36.1%) | *0.27* |
| Unstable housing | 59 (22.8%) | 79 (12.0%) | ***<0.001*** |
| Lived: |  |  | ***<0.01*** |
| Alone | 44 (16.2%) | 90 (12.9%) |  |
| With parents | 104 (38.4%) | 304 (43.4%) |  |
| With a partner (as a couple) | 48 (17.7%) | 170 (24.3%) |  |
| Other | 75 (27.7%) | 136 (19.4%) |  |

Data are mean (SD) or n (%).
