# Supplementary material for: Effects of a systematically offered social and preventive medicine consultation on training and health attitudes of young people not in employment, education or training (NEETs): An interventional study in France
Source: PLoS One. 2019 Apr 26;14(4):e0216226. doi: 10.1371/journal.pone.0216226 (PMC6485762; doi:10.1371/journal.pone.0216226)
Supplement: S2 Table — (DOCX) [file pone.0216226.s004.docx]

**S2 Table: Comparison of the lost to follow up participants’ baseline characteristics by group**

|  | **Intervention group (n=141)** | **Control group**  **(n=131)** | ***p*** |
| --- | --- | --- | --- |
| Age (years) | 21.1 (2.2) | 21.1 (2.0) | *0.71* |
| Gender |  |  | *0.72* |
| Female | 72 (51.1%) | 64 (48.9%) |  |
| Male | 69 (48.9%) | 67 (51.2%) |  |
| Origin |  |  | *0.75* |
| French, born to two French parents | 82 (58.2%) | 82 (62.6%) |  |
| French, born to foreign parent(s) | 44 (31.2%) | 37 (28.2%) |  |
| Foreigner | 15 (10.6%) | 12 (9.2%) |  |
| Level of education |  |  | *0.36* |
| Middle school | 19 (13.5%) | 16 (12.2%) |  |
| High school | 87 (61.7%) | 91 (69.5%) |  |
| Postsecondary | 35 (24.8%) | 24 (18.3%) |  |
| Difficulty reading French | 19 (13.5%) | 22 (16.8%) | *0.45* |
| Difficulty writing in French | 39 (27.7%) | 48 (36.6%) | *0.11* |
| No income | 67 (51.2%) | 54 (45.0%) | *0.33* |
| Had a partner | 48 (34.3%) | 39 (30.2%) | *0.48* |
| Unstable housing | 33 (24.6%) | 26 (20.8%) | *0.46* |
| Lived: |  |  | *0.99* |
| Alone | 23 (16.3%) | 21 (16.2%) |  |
| With parents | 53 (37.6%) | 51 (39.2%) |  |
| With a partner (as a couple) | 25 (17.7%) | 23 (17.7%) |  |
| Other | 40 (28.4%) | 35 (26.9%) |  |

Data are mean (SD) or n (%).
